# Comparison of 2-Aminobenzamide, Procainamide and *Rapi*Fluor-MS as Derivatizing Agents for High-Throughput HILIC-UPLC-FLR-MS N-glycan Analysis

**DOI:** 10.3389/fchem.2018.00324

**Published:** 2018-07-26

**Authors:** Toma Keser, Tamara Pavić, Gordan Lauc, Olga Gornik

**Affiliations:** ^1^Faculty of Pharmacy and Biochemistry, University of Zagreb, Zagreb, Croatia; ^2^Genos Glycoscience Research Laboratory, Zagreb, Croatia

**Keywords:** N-glycans, 2-aminobenzamide, procainamide, *Rapi*Fluor-MS, IgG, HILIC, fluorescence, mass spectrometry

## Abstract

Rising awareness of the universal importance of protein N-glycosylation governs the development of further advances in N-glycan analysis. Nowadays it is well known that correct glycosylation is essential for proper protein function, which emanates from its important role in many physiological processes. Furthermore, glycosylation is involved in pathophysiology of multiple common complex diseases. In the vast majority of cases, N-glycosylation profiles are analyzed from enzymatically released glycans, which can be further derivatized in order to enhance the sensitivity of the analysis. Techniques wherein derivatized N-glycans are profiled using hydrophilic interaction chromatography (HILIC) with fluorescence (FLR) and mass spectrometry (MS) detection are now routinely performed in a high-throughput manner. Therefore, we aimed to examine the performance of frequently used labeling compounds −2-aminiobenzamide (2-AB) and procainamide (ProA), and the recently introduced *Rapi*Fluor-MS (RF-MS) fluorescent tag. In all experiments N-glycans were released by PNGase F, fluorescently derivatized, purified by HILIC solid phase extraction and profiled using HILIC-UPLC-FLR-MS. We assessed sensitivity, linear range, limit of quantification (LOQ), repeatability and labeling efficiency for all three labels. For this purpose, we employed in-house prepared IgG and a commercially available IgG as a model glycoprotein. All samples were analyzed in triplicates using different amounts of starting material. We also tested the performance of all three labels in a high-throughput setting on 68 different IgG samples, all in duplicates and 22 identical IgG standards. In general, ProA labeled glycans had the highest FLR sensitivity (15-fold and 4-fold higher signal intensities compared to 2-AB and RF-MS respectively) and RF-MS had the highest MS sensitivity (68-fold and 2-fold higher signal intensities compared to 2-AB and ProA, respectively). ProA and RF-MS showed comparable limits of quantification with both FLR and MS detection, whilst 2-AB exhibited the lowest sensitivity. All labeling procedures showed good and comparable repeatability. Furthermore, the results indicated that labeling efficiency was very similar for all three labels. In conclusion, all three labels are a good choice for N-glycan derivatization in high-throughput HILIC-UPLC-FLR-MS N-glycan analysis, although ProA and RF-MS are a better option when higher sensitivity is needed.

## Introduction

Rising awareness of the vital role that N-glycosylation plays in protein functioning governs the need for further advances in N-glycan analysis. Nowadays it is well established that correct glycosylation is essential for proper protein function and stability. Therefore, it is not surprising that N-glycans exert their influence in many physiological processes and that aberrant N-glycosylation is linked to the pathophysiology of multiple common complex diseases (Gornik et al., 2012). Moreover, glycans have also been drawn into the spotlight in the biopharma industry, where they are denoted as a critical quality attribute for therapeutic proteins that are glycosylated, such as monoclonal antibodies or erythropoietin (Beck, 2013; Kauffman et al., 2016).

In general, there are three major approaches for N-glycan analysis–characterization of glycans on intact glycoproteins, characterization of glycopeptides or structural analysis of chemically or enzymatically released glycans (Mariño et al., 2010). In the vast majority of cases N-glycans are profiled after enzymatic release from the protein backbone (Tarentino et al., 1985). Because free glycans lack chromophore or fluorophore properties, and do not ionization well, they are often derivatized, to facilitate their separation or detection, and to enhance the sensitivity of the analysis. Such derivatized N-glycans are most commonly profiled using liquid chromatography (LC), mass spectrometry (MS) or a combination of both.

Released N-glycans are often derivatized using a fluorescent tag, which enables their optical detection in LC profiling. Some fluorescent labels can also improve ionization efficiency, which then facilitates MS detection. One of the most frequently used labeling compounds is 2-aminobenzamide (2-AB) (Ruhaak et al., 2010), which binds to the glycan via a reductive amination reaction. This label contains a primary amine group that reacts with the aldehyde group of the glycan, resulting in an imine, which is then reduced (by a reducing agent) to produce a stable secondary amine (Ruhaak et al., 2010). The advantage of this approach is the stoichiometric attachment of one molecule of label per one molecule of glycan, allowing the relative quantification of different glycans based on fluorescence intensity (Ruhaak et al., 2010). However, the major disadvantage of 2-AB is poor ionization efficiency, which hinders MS analysis.

Procainamide (4-amino-*N*-[2-(diethylamino)ethyl] benzamide; ProA) is another fluorescent tag, which uses the same mechanism as 2-AB to bind to the reducing end of a glycan, but it shows increased fluorescence and ionization performance (Klapoetke et al., 2010; Kozak et al., 2015). This can be explained by the fact that ProA contains a basic tertiary amine tail with high proton affinity, hence it exhibits higher sensitivity in positive mode ESI-MS (Kozak et al., 2015).

Recently, Waters introduced a new labeling compound, named *Rapi*Fluor-MS (RF-MS), which uses different chemistry from the aforementioned labels (Lauber et al., 2015). Namely, RF-MS contains a “rapid tagging functional group,” i.e., *N*-hydroxysuccinimide carbamate, which rapidly (within 5 min) modifies glycosylamine-bearing N-glycans after their enzymatic release, yielding a stable urea linkage. Apart from the rapid tagging group, RF-MS also contains a quinoline fluorophore (to provide the possibility of fluorescence detection) and a basic tertiary amine (to enhance positive mode electrospray ionization– as is the case for ProA).

Studies wherein N-glycans and other biomarkers are profiled using hydrophilic interaction chromatography with fluorescence detection (HILIC-UPLC-FLR), capillary electrophoresis laser-induced fluorescence (CE-LIF) or positive mode electrospray mass spectrometry (ESI-MS) are now routinely performed in a high-throughput manner (Li et al., 2011; Huffman et al., 2014; Schwedler et al., 2014; Kozak et al., 2015; Giorgetti et al., 2018). Here we present the comparative performance of three labeling compounds −2-AB, ProA and the recently introduced RF-MS - in the context of N-glycan profiling by HILIC-UPLC-FLR coupled to ESI-QTOF-MS. We assessed method sensitivity, linear range, limit of quantification (LOQ), repeatability and labeling efficiency for all three labels. For this purpose, we employed in-house prepared IgG and a commercially available IgG standard, as a model glycoprotein. We also tested the performance of all three labels in a high-throughput setting.

## Materials and methods

### IgG samples

Two different types of IgG samples were used for all experiments (except for the high-throughput test): in-house prepared IgG (denoted as isolated IgG), which was isolated from human plasma by affinity chromatography using protein G monoliths according to a previously published protocol (Pucić et al., 2011), and commercially available IgG (reagent grade, ≥95%, essentially salt-free, lyophilized powder, Sigma-Aldrich, St. Louis, MO, USA), denoted as standard IgG. Stock solutions were made and the concentration of IgG was determined by UV-Vis spectrophotometry at 280 nm. For the testing of 2-AB and ProA labels, the following amounts of standard and isolated IgG were prepared in triplicates (from the stock solutions): 0.05, 0.1, 0.5, 1, 5, 10, 25, 50, 75, 100, 250 and 500 μg. For the testing of RF-MS labeled glycans the following IgG amounts were prepared: 0.05, 0.1, 0.5, 1, 2, 4, 7.5, 15 and 30 μg (also from stock solutions in triplicates, for both standard and isolated IgG). A narrower concentration range was taken for RF-MS testing because the manufacturer stated that their protocol is designed for a glycoprotein quantity of 15 μg (Waters Corporation, 2017).

### IgG samples for the high-throughput test

For the high-throughput test, 68 different human plasma samples were used, all in duplicates. Additionally 22 identical standards were prepared from pooled plasma. Plasma was taken from healthy individuals and the study was approved by the Ethics Committee of the Faculty of Pharmacy and Biochemistry, University of Zagreb, Zagreb, Croatia. All subjects gave written informed consent in accordance with the Declaration of Helsinki. The plasma samples were randomized onto two different 96-well plates. IgG was isolated from the plasma samples using protein G monolithic plates as described previously (Pucić et al., 2011). Briefly, 90 μl of plasma was diluted 10x with PBS, applied to the protein G plate (BIA Separations, Ljubljana, Slovenia) and instantly washed. IgGs were eluted with 1 ml of 0.1 M formic acid and neutralized with 1 M ammonium bicarbonate. A NanoDrop (Thermo Fisher Scientific, Waltham, MA, USA) spectrophotometer was used to measure IgG concentration in each sample.

### Glycan analysis

#### 2-AB labeled N-glycans

Dried IgG samples were resuspended and denatured by incubation with 30 μl SDS (1.33% wt/vol; Invitrogen, Carlsbad, CA, USA) at 65°C for 10 min. Subsequently, 10 μl of 4% Igepal-CA630 (Sigma-Aldrich, St Louis, MO, USA) and 1.2 u PNGase F (Promega, Madison, WI, USA) in 10 μl 5 × PBS were added. The samples were incubated overnight at 37°C to allow release of N-glycans. The released N-glycans were labeled with 2-AB. The labeling mixture was freshly prepared by dissolving 2-AB (19.2 mg/ml; Sigma-Aldrich) and 2-picoline borane (44.8 mg/ml; Sigma-Aldrich) in a mixture of DMSO (Sigma-Aldrich) and glacial acetic acid (Merck, Darmstadt, Germany) (70:30, vol/vol). Labeling mixture (25 μl) was added to each N-glycan sample in the 96-well plate, which was then sealed using adhesive seal. Mixing was achieved by shaking for 10 min, followed by incubation at 65°C for 2 h. To each sample (75 μl), 700 μl of acetonitrile (ACN) (J. T. Baker, Phillipsburg, NJ, USA) was added. Free label and reducing agent were removed from the samples using HILIC solid-phase extraction (SPE). A GHP filter plate, 0.2 μm, (Pall Corporation, Ann Arbor, MI, USA) was used as the stationary phase. All wells were prewashed using 1 × 200 μl of ethanol/water (70:30, vol/vol) and 1 × 200 μl water, followed by equilibration using 1 × 200 μl of ACN/water (96:4, vol/vol). Solvent was removed by the application of a vacuum using a vacuum manifold (Millipore Corporation, Billerica, MA, USA). The samples were loaded into the wells, which were subsequently washed five times using 200 μl of ACN/water (96:4, vol/vol). Glycans were eluted with 2 × 90 μl of water and combined eluates were stored at −20°C until usage.

#### ProA labeled N-glycans

ProA labeled glycans were prepared in the same way as 2-AB labeled glycans. The only difference was that the labeling mixture contained 38.3 mg/ml ProA (procainamide hydrochloride, Sigma-Aldrich) instead of 19.2 mg/ml 2-AB (equimolar concentrations).

#### RF-MS labeled N-glycans

The Glycoworks *Rapi*Fluor-MS N-Glycan kit was obtained from Waters Corporation (Milford, MA, USA). The IgG samples were deglycosylated, labeled and purified by HILIC-SPE according to the manufacturer's protocol (Waters Corporation, 2017). The labeled glycans were stored at −20°C until usage.

### HILIC-UPLC-FLR-MS

Fluorescently labeled N-glycans were separated by HILIC on an Acquity H-class ultra-performance liquid chromatography (UPLC) instrument (Waters) consisting of a quaternary solvent manager, sample manager and a fluorescence detector (FLR, set with excitation and emission wavelengths 250 and 428 nm for 2-AB, 310 and 370 nm for ProA, and 265 and 425 nm for RF-MS, respectively), coupled with a Synapt G2-Si ESI-QTOF-MS system (Waters). The instrument was under the control of MassLynx v.4.1 software (Waters).

Labeled N-glycans were separated on a Waters bridged ethylene hybrid (BEH) Glycan chromatography column, 100 × 2.1 mm, 1.7 μm BEH particles, with 50 mmol/l ammonium formate, pH 4.4, as solvent A and ACN as solvent B. The separation method used a linear gradient of 75–62% acetonitrile (vol/vol) at flow rate of 0.4 ml/min in a 27 min analytical run. Samples were maintained at 10°C before injection and the separation temperature was 60°C. The injection volume was 30 μl of ACN/sample (75:25, vol/vol), which is 4.17% of the total 2-AB and ProA labeled samples and 7.5% of the total RF-MS labeled samples. The system was calibrated using an external standard of hydrolyzed and 2-AB, ProA or RF-MS labeled glucose oligomers from which the retention times for the individual glycans were converted to glucose units.

MS conditions were set as follows: positive ion mode, capillary voltage 3 kV, sampling cone voltage 30 V, source temperature 120°C, desolvation temperature 350°C, desolvation gas flow 800 l/h. Mass spectra were recorded from 500 to 3,000 m/z at a frequency of 1 Hz. MS/MS experiments were performed in a data-dependent acquisition (DAD) mode. Spectra were first acquired from 500 to 3,000 m/z and then three precursors with the highest intensities were selected for CID fragmentation (m/z 100 to 3,000 was recorded). A collision energy ramp was used for the fragmentation (LM CE Ramp Start 7 V, LM CE Ramp End 12 V, HM CE Ramp Start 105 V, HM CE Ramp End 115 V).

### Data processing and analysis

The chromatographic glycan peaks resulting from the UPLC-fluorescence analysis were processed with Empower 3 software (Waters) using an automated method with a traditional integration algorithm after which each chromatogram was manually corrected to maintain the same intervals of integration for all the samples. The chromatograms were all separated in the same manner into 22 peaks, if their S/N was above 10. Total area normalization was performed, where the area of each glycan peak was divided by the total area of the corresponding chromatogram. Glycan peaks were analyzed on the basis of their elution positions and measured in glucose units then compared to reference values in the “GlycoStore” database (available at: https://glycostore.org/) for structure assignment (Campbell et al., 2008; Abrahams et al., 2018).

LaCyTools (version 1.0.1, b20171027a) (Jansen et al., 2016) was used for automated relative quantification of the MS data. Before processing, LC-MS files were converted to mzXML files. Chromatograms were aligned based on the six most abundant glycan signals. Targeted peak integration was performed on singly, doubly and triply charged species. Protonated, sodium, potassium and ammonium adducts were observed. Signals were integrated to include at least 85% of the theoretical isotopic pattern. The actual presence of a glycan was assessed based on the mass accuracy (between −20 and 20 ppm), the deviation from the theoretical isotopic pattern (IPQ; below 25%), and the S/N (above 10) of an integrated signal. Total area normalization was performed for each sample.

Data was analyzed using Microsoft Excel. Linearity was estimated using the “least squares” method and coefficients of determination (*R*^2^) were calculated, first for the whole range of measured N-glycans from different IgG concentrations, and then for the ranges where the highest concentrations were consecutively excluded (Supplementary Figure 1, Supplementary Tables 1–3). The ranges where *R*^2^ became ≥0.99 (rounded to 2 decimal places) were taken as the linear range. Repeatability was estimated using replicates of the same sample–calculating the coefficient of variation (CV, the ratio of the standard deviation to the mean), and using duplicates of different samples–calculating *R*^2^ (square of the Pearson correlation coefficient).

## Results

### HILIC-UPLC-FLR-MS analysis of 2-AB, ProA and RF-MS labeled IgG N-glycans

**Figure 1:**
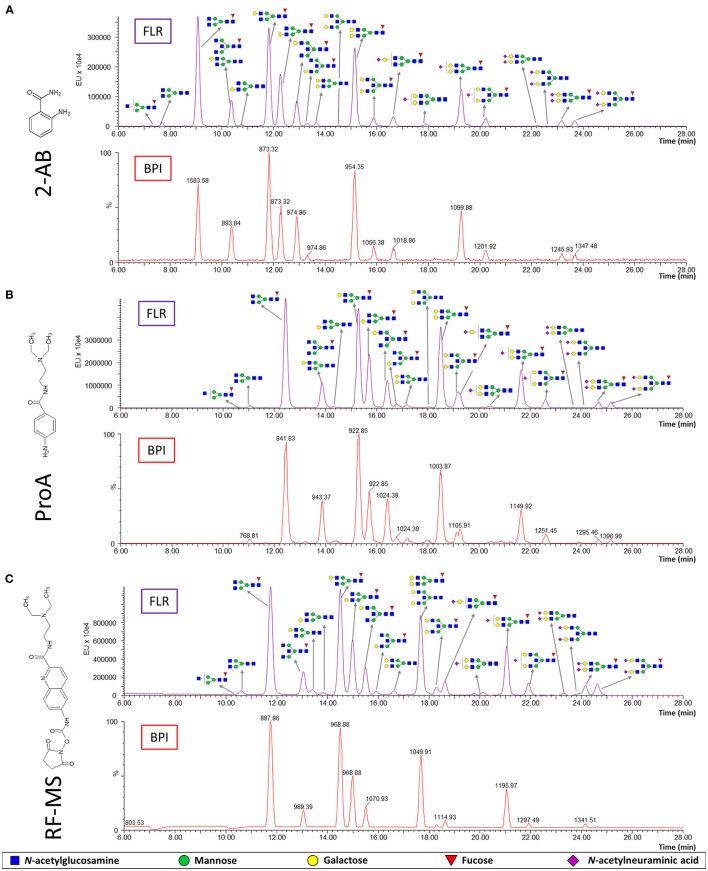
Fluorescence (FLR) and base peak intensity (BPI) chromatograms obtained by HILIC-UPLC-FLR-MS analysis of IgG N-glycans labeled with three different labels: 2-AB **(A)**, ProA **(B)** and RF-MS **(C)**. The structure and m/z of the most abundant glycans are shown for each peak.

N-glycans were released from IgG samples with PNGase F, labeled with one of the three fluorescent labels, purified with HILIC-SPE and analyzed with HILIC-UPLC-FLR-MS. Therefore, FLR and MS signals were obtained for each label (Figure 1). All glycan structures were determined and confirmed by glucose units (compared to reference values in the “GlycoStore” database) and MS/MS analysis (Supplementary Figure 2). Chromatographic separation of the IgG N-glycans was satisfactory and comparable with all three labels (Figure 1). Glycans labeled with ProA and RF-MS showed higher retention times compared to 2-AB labeled glycans. The only differences in chromatographic separation were as follows: FA2B and A2[6]G1 (see Supplementary Table 4 for the explanation of the glycan nomenclature) co-eluted when labeled with 2-AB or ProA, but eluted separately when labeled with RF-MS; A2BG2 and FA2G2 co-eluted when labeled with RF-MS, but eluted separately when labeled with 2-AB or ProA; separation between FA2BG2 and FA2G1S1 was poorer with ProA.

### Linear range and sensitivity

**Figure 2:**
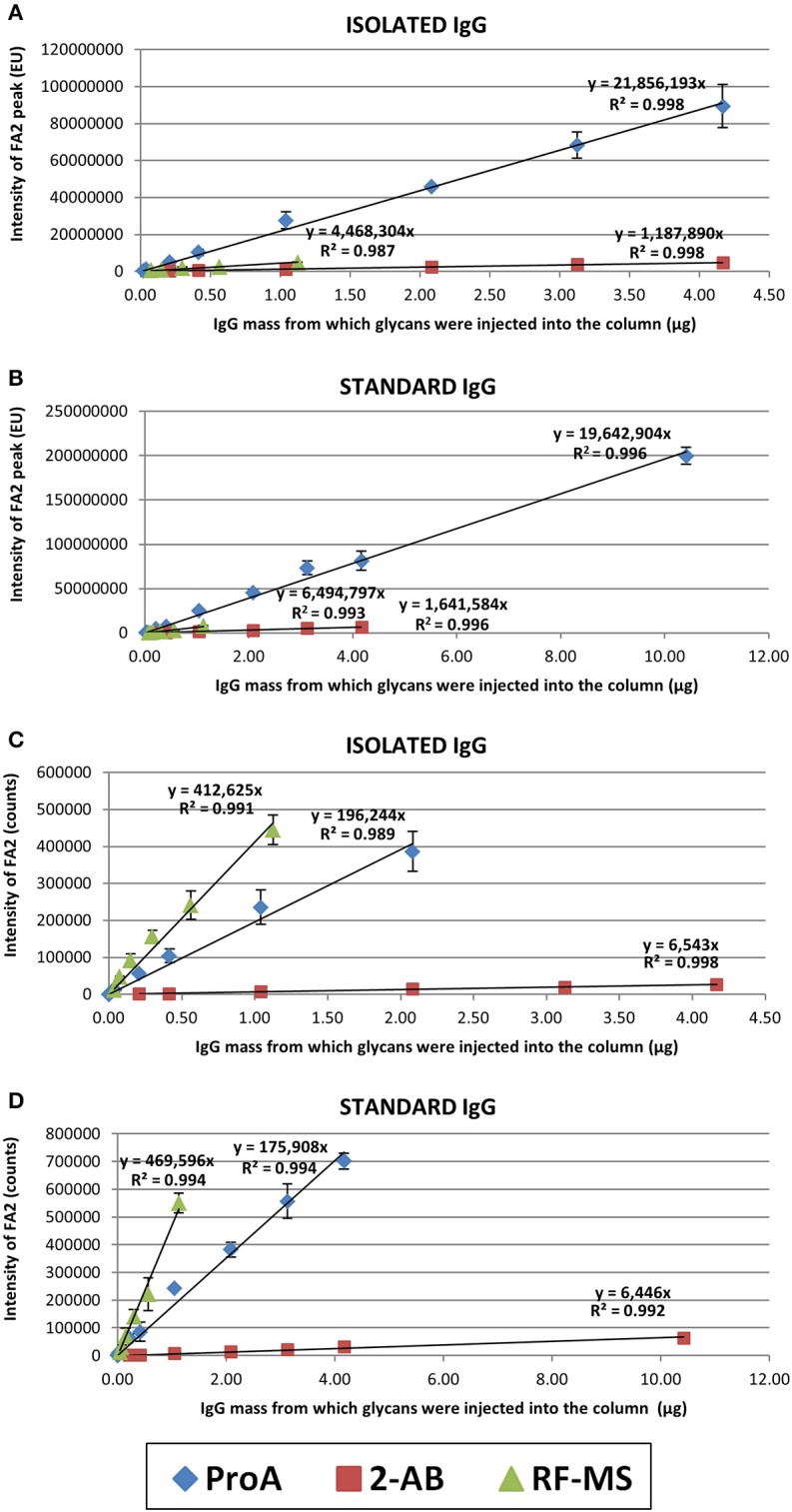
Linear range of detection for the FA2 glycan labeled with ProA (diamond), 2-AB (square) or RF-MS (triangle). Shown are both the FLR signal, for the isolated **(A)** and the standard **(B)** IgG sample, and the MS signal, for the isolated **(C)** and the standard **(D)** IgG sample. Each concentration of each sample was analyzed in triplicate and error bars represent the standard deviation of the triplicates.

The linear range of detection was determined for each label for both isolated (in-house prepared) and standard (commercial) IgG samples, for both FLR and MS signals (Figure 2). Linearity was estimated by using the “least squares” method (see Materials and methods for more details). RF-MS labeled glycans had a narrower linear range for both FLR and MS signals, compared to 2-AB and ProA labeled glycans (Figure 2, Supplementary Tables 1–3). ProA labeled glycans showed a wider linear range for the FLR signal, while 2-AB labeled glycans showed a wider linear range for the MS signal. Differences in sensitivity between the three labels were calculated as the ratios of slopes of the linear range equations (Table 1). 2-AB labeled glycans showed the lowest sensitivity, for both FLR and MS signals. ProA labeled glycans had the highest FLR sensitivity (on average 15.2 times higher signal than 2-AB and 4.0 times higher signal than RF-MS), while RF-MS had the highest MS sensitivity (on average 68.0 times higher signal than 2-AB and 2.4 higher signal than ProA).

**Table 1:** Differences in sensitivity in FLR and MS signal between 2-AB, ProA and RF-MS.

**Signal**	Slope ratio^*^	**Isolated IgG**	**Standard IgG**	**Average**
FLR	ProA vs. 2AB	18.4	12.0	15.2
	ProA vs. RapiFluor	4.9	3.0	4.0
	RapiFluor vs. 2AB	3.8	4.0	3.9
MS	ProA vs. 2AB	30.0	27.3	28.6
	RapiFluor vs. ProA	2.1	2.7	2.4
	RapiFluor vs. 2AB	63.1	72.9	68.0

* *Differences in sensitivity between the three labels were calculated as the ratios of the slopes of linear range equations*.

### Relative quantification

To make measurements across samples comparable, total area normalization was performed (see Materials and methods for more details). Coefficients of variation (CVs) were calculated for each label, for the isolated and the standard IgG sample, for both FLR and MS signals in ranges from the LOQ (based on the quality parameters in Materials and methods) to the maximum of the linear range (Table 2). Although MS can measure the signal of different coeluting glycan structures separately, the signals of coeluting structures were summed for the sake of easier comparison with FLR. Quantification of IgG N-glycans with FLR detection was very good and comparable for all three fluorescent labels (average CVs of all glycan peaks were 3–4.2%). The minimal starting amount of IgG required for reliable quantification of individual glycans using FLR detection was 10 μg for 2-AB, 1–5 μg for ProA and 1 μg for RF-MS. This corresponds to 0.42 μg of IgG for 2-AB, 0.04–0.21 μg for ProA and 0.08 μg for RF-MS, from which glycans were injected into the column (glycans were not concentrated before the injection in order to eliminate the influence of drying). MS detection provided poorer quantification of IgG N-glycans (average CVs of all glycan peaks were 9.6–18.6%). ProA labeled glycans that contain sialic acid showed higher CVs with MS detection compared to 2-AB and RF-MS labeled glycans (Table 2). The minimal starting amount of IgG required for reliable quantification of individual glycans with MS detection was 10 μg for 2-AB, 1 μg for ProA and 0.5 μg for RF-MS. This corresponds to 0.42 μg of IgG for 2-AB and 0.04 μg for ProA and RF-MS, from which glycans were injected to the column.

**Table 2:** Differences in relative quantification of N-glycans labeled with 2-AB, ProA or RF-MS.

	**Signal**	**FLR**						**MS**					
	**Label**	**2-AB**		**ProA**		**RF-MS**		**2-AB**		**ProA**		**RF-MS**	
	**IgG**	**Isolated**	**Standard**	**Isolated**	**Standard**	**Isolated**	**Standard**	**Isolated**	**Standard**	**Isolated**	**Standard**	**Isolated**	**Standard**
	**Starting range (**μ**g)**^a^	**10–100**	**10–100**	**1–100**	**5–250**	**1–15**	**1–15**	**10–100**	**10–250**	**1–50**	**1–100**	**0.5–15**	**0.5–15**
	**Injection range (**μ**g)**^b^	**0.42–4.17**	**0.42–4.17**	**0.04–4.17**	**0.21–10.42**	**0.08–1.13**	**0.08–1.13**	**0.42–4.17**	**0.42–10.42**	**0.04–2.08**	**0.04–4.17**	**0.04–1.13**	**0.04–1.13**
CV (%)	FA1	10.7	20.9	9.0	22.7	15.3	31.8	38.9	41.1	16.1	34.2	62.5	86.8
	A2	3.6	3.8	5.0	3.5	5.6	4.5	27.8	12.8	17.8	26.6	9.7	17.7
	FA2	0.8	0.9	1.2	0.6	0.6	0.5	4.7	2.7	5.4	5.4	8.7	14.9
	FA2B + A2[6]G1	0.4	0.7	1.6	1.6	1.3	1.2	3.6	23.0	6.8	3.5	9.7	13.2
	A2[3]G1	4.5	4.8	3.2	2.9	2.2	3.3	38.8	29.6	13.0	23.3	9.8	10.7
	FA2[6]G1	0.7	0.5	1.0	0.4	0.5	0.2	2.6	2.7	4.5	6.1	2.6	4.2
	FA2[3]G1	0.4	0.5	0.8	0.5	0.4	0.4	1.8	2.4	1.5	2.0	4.2	8.2
	FA2[6]BG1	0.4	0.8	1.8	2.3	0.6	0.4	4.3	2.0	7.8	6.7	3.4	3.9
	FA2[3]BG1	2.7	2.6	11.9	10.8	2.4	2.6	14.5	10.0	10.2	7.0	6.5	6.1
	A2G2	2.7	3.8	4.8	7.3	2.0	2.5	15.0	13.5	11.2	20.4	5.9	9.4
	FA2G2 + A2BG2	0.8	0.8	1.1	0.9	0.5	0.4	2.2	3.2	3.5	5.1	2.8	3.5
	FA2BG2	3.3	2.1	2.6	2.6	1.4	2.7	3.7	4.3	9.3	8.7	4.2	5.3
	FA2G1S1	1.7	2.0	3.4	2.9	1.2	1.6	5.2	3.7	12.6	18.3	4.5	6.4
	A2G2S1	4.4	4.0	2.4	2.1	2.3	3.5	9.9	7.1	21.1	32.4	6.9	8.9
	FA2G2S1	0.9	1.2	1.2	0.8	0.6	0.6	3.9	4.5	10.6	14.7	6.0	10.6
	FA2BG2S1	3.1	2.3	3.1	2.2	1.6	1.5	6.2	4.6	11.6	20.9	6.7	13.3
	A2G2S2	8.0	8.7	9.6	2.8	3.2	4.3	16.2	14.4	15.2	30.4	12.0	14.0
	A2BG2S2	16.3	13.4	13.7	10.3	10.9	6.2	17.0	31.5	47.0	56.8	8.2	16.3
	FA2G2S2	2.1	2.9	3.2	2.7	2.5	2.3	17.9	7.6	13.8	25.6	9.1	17.0
	FA2BG2S2	2.4	3.2	3.5	3.5	4.2	2.6	9.6	6.3	10.9	23.2	9.3	19.1
	Average	3.5	4.0	4.2	4.2	3.0	3.7	12.2	11.3	12.5	18.6	9.6	14.5

*Repeatability was estimated using CVs, which were calculated in the range from the LOQ to the maximum of linear range*.

a *Starting amounts of IgG from which N-glycans were released (each amount was done in triplicate)*.

b *Amounts of IgG from which glycans were injected into the column (from the LOQ to the maximum of linear range, each amount was done in triplicate). The starting and injection ranges are different because only a part of the total glycan sample volume was injected into the column for analysis (samples were not concentrated before the injection to eliminate the effect of drying on the results)*.

**Figure 3:**
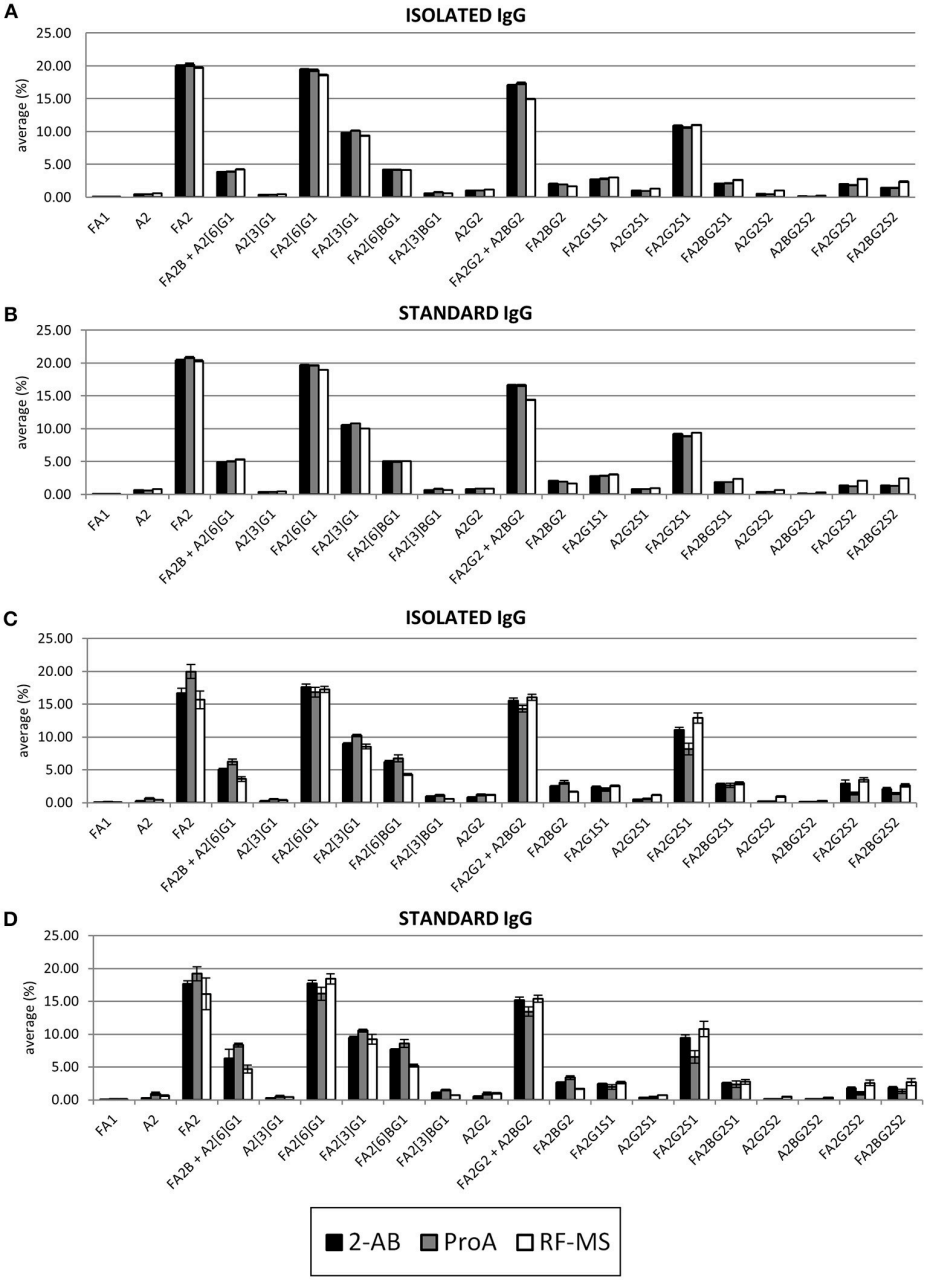
Comparison of glycan profiles obtained with 2-AB (black), ProA (gray) and RF-MS (white) labels, with FLR detection for the isolated **(A)** and the standard **(B)** IgG samples, and with MS detection for the isolated **(C)** and the standard **(D)** IgG samples. The height of the bars represents the average of the relative amount of the specific glycan within the range from the LOQ to the maximum of linear range, while error bars represent the standard deviation of the same range. The relative amount of each glycan was calculated by total area normalization.

Furthermore, glycan profiles obtained with each label were compared (Figure 3). 2-AB and ProA labeled IgG glycans showed almost identical profiles using FLR detection, whereas the profile obtained with RF-MS label using FLR detection showed a decrease in some galactosylated glycans without sialic acids and an increase in glycans with sialic acids, compared to 2-AB and ProA profiles. Profiles obtained by MS detection were slightly more variable. Again, profiles obtained with RF-MS using MS detection had the highest relative amount of glycans with sialic acids. In contrast, profiles obtained with ProA, using MS detection had the lowest amount of glycans with sialic acids, while some neutral glycans, except for FA2[6]G1 and FA2G2+A2BG2, were increased.

### Labeling efficiency

Free, unlabeled glycans were detected by MS in each sample (Supplementary Figure 3). Differences in labeling efficiency were estimated based on the ratios of the absolute amounts of unlabeled major glycans (FA2, FA2G1, FA2G2, FA2G2S1, FA2G2S2, FA2BG2S2) between the samples labeled with different labels, for the same initial IgG amounts (Supplementary Tables 5–8). Levels of free unlabeled IgG glycans were practically the same for all three labels in the samples obtained with the same starting amount of IgG the average ratio of unlabeled glycans between 2-AB and ProA labeled samples was 1.0 for the isolated IgG samples and 1.1 for the standard IgG samples, and the average ratio of unlabeled glycans between ProA and RF-MS labeled samples was 1.0 for the isolated IgG samples and 1.2 for the standard IgG samples. This indicates that the labeling efficiency is very similar for all three labels.

### The high-throughput test

In order to test the performance of each label in a high-throughput manner, 68 different IgG samples were used, all in duplicates. Additionally 22 identical IgG standards were prepared. All the samples were randomized three times, once for each label, onto two different 96-well plates in the amounts which are in the linear rage of the corresponding label. N-glycans were released from IgG, labeled with 2-AB, ProA or RF-MS, purified with HILIC-SPE and analyzed with HILIC-UPLC. FLR was chosen for the detection, because it proved to be better than MS for 2-AB, ProA, and RF-MS labeled IgG N-glycan quantification, as shown previously (Table 2). To make measurements across samples comparable, total area normalization was performed (see Materials and methods for more details). Repeatability was estimated by calculating the CVs of 22 identical IgG standards and by calculating coefficients of determination (*R*^2^) of duplicates of 68 different samples (Table 3). 2-AB labeled glycans had the highest average CVs of the replicates, of all glycan peaks (7.8%, compared to 4.4% for ProA and 7.0% for RF-MS). However, they also had the highest average *R*^2^ of the duplicates, of all glycan peaks (95.6%, compared to 92.8% for ProA and 87.8 % for RF-MS). Altogether, all three labels presented similar repeatability, although RF-MS labeled glycans showed noticeably poorer repeatability of some disialylated glycans (A2BG2S2, FA2G2S2, and FA2BG2S2).

**Table 3:** Repeatability assessment using the high-throughput test.

	**CV of replicates (%)**			***R***^**2**^ **of duplicates (%)**		
**Glycan**	**2-AB**	**ProA**	**RF-MS**	**2-AB**	**ProA**	**RF-MS**
FA1	15.0	5.2	9.0	94.9	99.4	98.6
A2	13.5	2.9	2.8	98.8	99.4	98.8
FA2	1.9	1.6	2.7	99.5	99.2	97.0
FA2B + A2[6]G1	4.4	2.0	2.2	99.1	98.3	98.6
A2[3]G1	11.4	4.6	1.8	99.0	95.2	98.8
FA2[6]G1	1.6	0.7	2.4	95.6	98.3	94.1
FA2[3]G1	2.0	0.8	2.2	99.2	98.6	96.4
FA2[6]BG1	1.5	1.4	1.7	99.5	99.2	99.4
FA2[3]BG1	6.0	8.7	1.7	95.8	52.1	87.3
A2G2	9.9	13.2	1.9	99.6	88.7	98.7
FA2G2 + A2BG2	1.3	1.5	1.9	98.7	98.8	99.1
FA2BG2	4.3	3.6	1.1	90.9	92.0	98.0
FA2G1S1	1.5	2.5	2.1	98.9	97.2	98.1
A2G2S1	13.6	5.5	2.3	94.6	93.8	95.6
FA2G2S1	2.1	2.2	3.5	99.2	98.4	95.6
FA2BG2S1	8.7	3.6	12.1	93.1	95.2	68.6
A2G2S2	27.1	5.9	10.1	93.7	79.1	92.0
A2BG2S2	16.0	8.7	22.9	70.8	86.1	64.9
FA2G2S2	5.5	6.1	21.3	96.9	93.9	57.5
FA2BG2S2	7.8	7.6	35.2	93.5	92.2	18.8
Average	7.8	4.4	7.0	95.6	92.8	87.8

*Repeatability was estimated by calculating CVs of 22 identical IgG standards and by calculating R^2^ of duplicates of 68 different samples*.

## Discussion

Here we compared the performance of three labeling compounds −2-AB, ProA and RF-MS – for N-glycan profiling by HILIC-UPLC-FLR-MS. The method which we used for labeling glycans with 2-AB and ProA is done with a traditional deglycosylation procedure, where the glycoprotein sample is incubated overnight with PNGase F. Combined with this process is a 2 h labeling step based on the reductive amination of aldehyde termini that form on N-glycans only after they hydrolyze from their glycosylamine forms. The RF-MS method provides more throughput glycoproteins are deglycosylated in 5 min to produce N-glycosylamines. Glycosylamines are then rapidly (within 5 min) labeled with RF-MS before they hydrolyze to reducing, aldehyde terminated glycans.

Labeled glycans were purified with HILIC-SPE and analyzed with HILIC-UPLC-FLR-MS. Relative quantification of IgG N-glycans with FLR detection was very good and comparable for all three fluorescent labels, while MS detection provided poorer quantification, although it was slightly more sensitive than the FLR. A reason for this could be that in ESI-MS glycans form adducts with protons, sodium, potassium and ammonium, which splits the glycan signal into multiple peaks and makes the quantification less robust (Grünwald-Gruber et al., 2017). However, MS can be used to measure the ratio of different glycans which coelute in the same peak. Therefore, these two detection techniques are complementary.

RF-MS labeled glycans had a narrower linear range for both the FLR and MS signal, compared to 2-AB and ProA labeled glycans, because the GlycoWorks *Rapi*Fluor-MS N-Glycan Kit is optimized for the release, labeling and extraction of N-glycans from 15 μg of glycoprotein. Changes in the glycoprotein quantity affect the PNGase F to substrate ratio as well as the molar excess of labeling reagent (RF-MS is a highly reactive, primary/secondary amine labeling reagent) which potentially results in a low yield of the labeled glycans (Waters Corporation, 2017). The isolated IgG samples had a narrower linear range than the standard IgG samples (Figure 2). This could be explained by the fact that standard IgG is a commercial IgG of reagent grade, essentially salt-free, while isolated IgG was prepared in-house in a high-throughput manner using a 96-well protein G monolithic plate. Therefore, isolated IgG presumably contains remains of salts from the buffers which were used during the IgG isolation procedure or maybe some other contamination, which could interfere with the sample preparation or with the analysis procedure.

2-AB labeled glycans showed the lowest sensitivity, for both the FLR and MS signal. 2-AB does not contain a basic tertiary amine tail with high proton affinity (in comparison to ProA and RF-MS), which can explain its lower ionization performance. ProA labeled glycans had the highest FLR sensitivity and RF-MS had the highest MS sensitivity. The higher sensitivity of ProA and RF-MS labeled glycans in MS enabled detection of seven additional minor N-glycan structures, which were barely or not detectable with 2-AB (Supplementary Table 9).

The only difference in methodology between 2-AB and ProA is the label itself, so the observed differences are the direct consequence of the labeling agent. By contrast, RF-MS methodology uses different enzymatic release and labeling chemistry, so we cannot conclude that the observed differences are influenced only by the RF-MS label. This can also explain why the glycan profiles obtained with 2-AB and ProA, using FLR detection are almost identical, while the glycan profile obtained with RF-MS showed an increase in glycans with sialic acids.

Differences in labeling efficiency between 2-AB, ProA and RF-MS were estimated based on the ratios of the absolute amounts of unlabeled glycans. Levels of the free unlabeled IgG glycans were very similar for all three labels in the samples obtained with the same starting amount of IgG, which suggests that the labeling efficiency is very similar for all three labels. This also points out that the labeling efficiency is not the cause of differences observed between the labels, despite the fact that RF-MS has a different labeling chemistry. The absolute labeling efficiency for each label could not be determined in this experiment because of the different MS response factors between unlabeled glycans and glycans labeled with different labels.

We also tested the repeatability of the 2-AB, ProA, and RF-MS procedures in a high-throughput setting. Altogether, all three labels demonstrated similar repeatability, although some disialylated N-glycans showed increased variability when labeled with RF-MS. A possible cause for this could be that RF-MS has the narrowest linear range and maybe some samples had IgG amounts that were larger than the upper limit of the linear range, although the IgG concentration was checked for each sample.

To put our research into context, we examined available literature on comparisons of the herein studied labels. In a study by Pabst et al. (2009) multiple fluorescent labels were tested for their performance in chromatographic and MS profiling of derivatized N-glycans. ProA showed double the fluorescence intensity of 2-AB in NP-HPLC, but the separation of different glycan species was unsatisfactory. The authors also compared label performance through different MS techniques (positive and negative mode MALDI-TOF-MS and ESI-MS), but found no significant differences in sensitivity between the labels. However, sample preparation differed between 2-AB and ProA labeled glycans, which could have affected the obtained results. Moreover, when the ProA labeled samples were measured online in positive mode ESI-MS, with a more concentrated formic acid, four times higher signal intensities were observed. This finding suggests that further optimization could have led to better method sensitivity.

Another study by Klapoetke et al. (2010) investigated ProA and 2-AB performance in HPLC-FLR and ESI-QTOF-MS. Both labels showed similar fluorescence intensities in chromatographic glycan profiling. In the case of 2-AB labeled glycans, better separation of closely eluting peaks was achieved, while ProA enabled the detection of minor peaks. In general, both labels showed comparable results in relative quantification of major glycans. When examining label performances in HPLC-ESI-QTOF-MS N-glycan profiling, ProA demonstrated that it has comparable MS and fluorescence sensitivity. On the contrary, the MS sensitivity of 2-AB was much lower than for fluorescence. To summarize, ProA improved ESI ionization efficiency by 10–50-fold.

Kozak et al. (2015) employed a system similar to ours–profiling of 2-AB and ProA derivatized N-glycans by HILIC-UPLC coupled to positive mode ESI-MS. Comparable N-glycan chromatographic profiles were obtained for samples derivatized with both labels, though ProA showed approximately 3-fold higher fluorescence signal intensities. However, positive mode ESI-MS signal intensities for ProA labeled glycans were up to 30-fold higher than their 2-AB labeled counterparts.

A study by Lauber et al. (2015), where the new label, RF-MS, was introduced also compared its HILIC-UPLC and ESI-MS performance to the conventional labels −2-AB and ProA. RF-MS showed 14-fold higher fluorescence and 160-fold higher MS signal intensities than 2-AB. When comparing RF-MS performance to the ProA, it was extrapolated from literature data that RF-MS could provide a 14-fold higher fluorescence sensitivity and a 3-fold higher gain in MS sensitivity. Since both ProA and RF-MS contain a tertiary amino group, it was postulated by the authors that the potential superior ionization of RF-MS labeled glycans originates from the higher hydrophobicity of RF-MS.

Lastly, Zhou et al. (2017) comprehensively assessed six different derivatization methods (2-AB, ProA, aminoxyTMT, RF-MS, reduction and reduction with permethylation) and their analysis on LC-MS and nano LC-MS systems. Different LC columns were also investigated to determine their compatibility with different derivatization methods. The RF-MS method enabled the shortest sample preparation time, while permethylation was the only method that eliminated sialic acid loss and rearrangement. For neutral glycans, RF-MS provided the highest MS signal, while permethylation exhibited a significant advantage in increasing MS intensity and structural stability of sialylated glycans. ProA showed slightly lower MS signal than RF-MS, while 2-AB had the lowest signal (only slightly better than reduced native glycans).

In our study, we observed 30-fold higher ESI ionization efficiency for ProA in comparison with 2-AB labeled glycans, which is concordant with studies by Klapoethe et al. and Kozak et al. Furthermore, we obtained the highest ESI ionization efficiency with RF-MS, which is concordant with the study by Zhou et al. On the contrary, we observed 15-fold higher FLR signals for ProA in comparison to 2-AB, while other studies reported similar or only slightly higher FLR signals for ProA.

To the best of our knowledge, our study is so far the most detailed and extensive comparison between 2-AB, ProA, and RF-MS and the first experimental comparison of FLR signal intensity between ProA and RF-MS. It was done on more than 180 samples for each label, where methods' sensitivity, linear range, LOQ, repeatability and labeling efficiency were assessed. This is the first study that tested the performance of the labels in a high-throughput setting, where all three of them showed comparable repeatability. Taking into account all presented data, we conclude that all three labels represent a good choice for N-glycan derivatization in high-throughput HILIC-UPLC-FLR-MS glycan profiling, although ProA and RF-MS are a better choice when higher sensitivity is needed.

## Ethics statement

This study was carried out in accordance with the recommendations of the Declaration of Helsinki - Recommendations Guiding Physicians in Biomedical Research Involving Human Subjects, and regulations of the Republic of Croatia: Pravilnik o dobroj kliničkoj praksi (NN 143/98) and Zakon o zaštiti prava pacijenata (NN 169/04). The protocol was approved by the Faculty of Pharmacy and Biochemistry Research Ethics Committee. All subjects gave written informed consent in accordance with the Declaration of Helsinki.

## Author contributions

TK conceived the study and designed it with TP and OG. OG and GL supervised the study. TK and TP participated in data acquisition, collection and analysis. All authors participated in interpretation. TK and TP drafted the manuscript. OG and GL critically revised the manuscript for intellectual content. All authors approved the final version of the manuscript.

### Conflict of interest statement

GL is founder and owner of Genos Ltd, a company that specializes in high-throughput glycomics and has several patents in this field. The remaining authors declare that the research was conducted in the absence of any commercial or financial relationships that could be construed as a potential conflict of interest.
