# Supplementary material for: Comparison of 2-Aminobenzamide, Procainamide and RapiFluor-MS as Derivatizing Agents for High-Throughput HILIC-UPLC-FLR-MS N-glycan Analysis
Source: Front Chem. 2018 Jul 26;6:324. doi: 10.3389/fchem.2018.00324 (PMC6070730; doi:10.3389/fchem.2018.00324)
Supplement: Supplementary file 1 [file Data_Sheet_1.DOCX]

Supplementary Material

Comparison of 2-aminobenzamide, procainamide and *Rapi*Fluor-MS as derivatizing agents for high-throughput HILIC-UPLC-FLR-MS N-glycan analysis

**Toma Keser^1*^, Tamara Pavić^1^, Gordan Lauc^1,2^, Olga Gornik^1^**

^1^ Faculty of Pharmacy and Biochemistry, University of Zagreb, Zagreb, Croatia

^2^ Genos Glycoscience Research Laboratory, Zagreb, Croatia

*** Correspondence:** Toma Keser: [tkeser@pharma.hr](mailto:tkeser@pharma.hr)

**Supplementary Table 1** Estimation of the linear range for 2-AB labeled FA2 by using the "least squares" method. Highlighted are cells in the range where R^2^ became ≥ 0.99 (rounded to 2 decimal places).

|  | **RANGE of IgG masses from which glycans were injected into the column (μg)** | | | | | |  |
| --- | --- | --- | --- | --- | --- | --- | --- |
|  | 0.42-20.83 | 0.42-10.42 | 0.42-4.17 | 0.42-3.13 | 0.42-2.08 | 0.42-1.04 |  |
| **R^2^** | **2-AB** isloated FLR | 0.22 | 0.67 | **1.00** | 1.00 | 1.00 | 1.00 |
|  | **2-AB** standard FLR | 0.97 | 0.88 | **1.00** | 1.00 | 0.99 | 0.99 |
|  | **2-AB** isolated MS | 0.20 | 0.64 | **1.00** | 1.00 | 1.00 | 0.98 |
|  | **2-AB** standard MS | 0.98 | **0.99** | 1.00 | 1.00 | 1.00 | 0.99 |

**Supplementary Table 2** Estimation of the linear range for ProA labeled FA2 by using the "least squares" method. Highlighted are cells in the range where R2 became ≥ 0.99 (rounded to 2 decimal places).

|  | | | **RANGE of IgG masses from which glycans were injected into the column (μg)** | | | | | | | | | | | | | | | |
| --- | --- | --- | --- | --- | --- | --- | --- | --- | --- | --- | --- | --- | --- | --- | --- | --- | --- | --- |
|  |  |  | 0.04-20.83 | | 0.04-10.42 | | 0.04-4.17 | | 0.04-3.13 | | 0.04-2.08 | | 0.04-1.04 | | 0.04-0.42 | | 0.04-0.21 | |
| **R^2^** | | **ProA** isloated FLR | 0.39 | | 0.62 | | **1.00** | | 1.00 | | 0.99 | | 1.00 | | 1.00 | | 1.00 | |
|  |  | **ProA** standard FLR | 0.98 | | **1.00** | | 0.99 | | 1.00 | | 1.00 | | 0.99 | | 0.99 | | 1.00 | |
|  |  | **ProA** isolated MS | 0.43 | | 0.79 | | 0.96 | | 0.98 | | **0.99** | | 1.00 | | 1.00 | | 1.00 | |
|  |  | **ProA** standard MS | 0.92 | | 0.97 | | **0.99** | | 0.99 | | 0.99 | | 0.99 | | 0.97 | | 0.99 | |

**Supplementary Table 3** Estimation of the linear range for RF-MS labeled FA2 by using the "least squares" method. Highlighted are cells in the range where R2 became ≥ 0.99 (rounded to 2 decimal places).

|  | **RANGE of IgG masses from which glycans were injected into the column (μg)** | | | | |  |
| --- | --- | --- | --- | --- | --- | --- |
|  | 0.04-2.25 | 0.04-1.13 | 0.04-0.56 | 0.04-0.3 | 0.04-0.15 |  |
| **R^2^** | **RF-MS** isloated FLR | 0.95 | **0.99** | 0.96 | 1.00 | 0.99 |
|  | **RF-MS** standard FLR | 0.98 | **0.99** | 0.99 | 1.00 | 0.99 |
|  | **RF-MS** isolated MS | 0.96 | **0.99** | 0.98 | 0.99 | 0.99 |
|  | **RF-MS** standard MS | 0.98 | **0.99** | 0.99 | 1.00 | 0.99 |

**Supplementary Table 4** Structure and description of the studied IgG N-glycans.

| **STRUCTURE^a^** | **DESCRIPTION** |
| --- | --- |
| 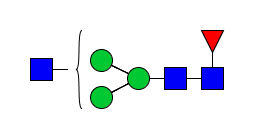 | **FA1** |
| 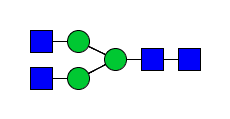 | **A2** |
| 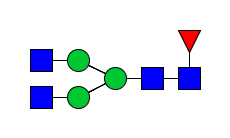 | **FA2** |
| 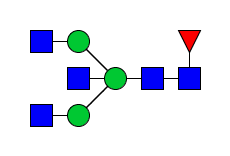 | **FA2B** |
| *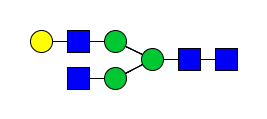* | **A2[6]G1** |
| 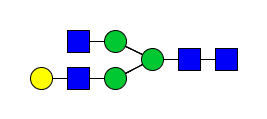 | **A2[3]G1** |
| 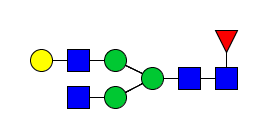 | **FA2[6]G1** |
| 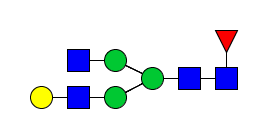 | **FA2[3]G1** |
| 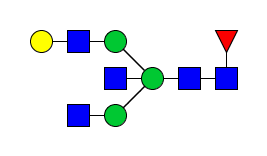 | **FA2[6]BG1** |
| 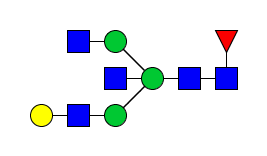 | **FA2[3]BG1** |
| 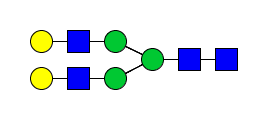 | **A2G2** |
| 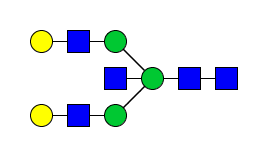 | **A2BG2** |
| 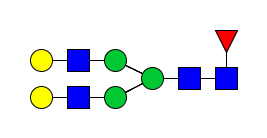 | **FA2G2** |
| 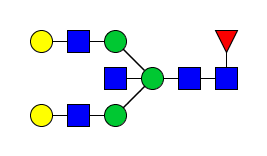 | **FA2BG2** |
| 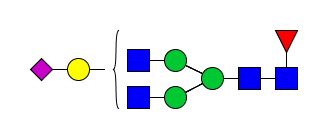 | **FA2G1S1** |
| 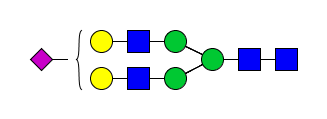 | **A2G2S1** |
| 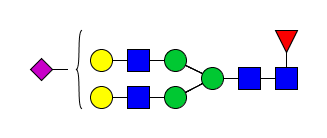 | **FA2G2S1** |
| 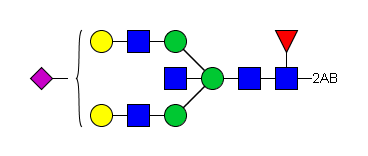 | **FA2BG2S1** |
| 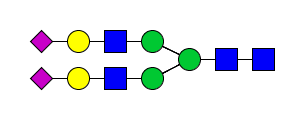 | **A2G2S2** |
| 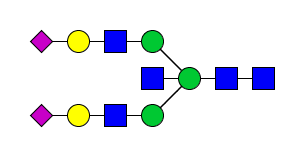 | **A2BG2S2** |
| 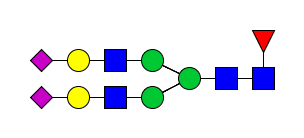 | **FA2G2S2** |
| 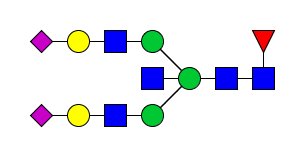 | **FA2BG2S2** |

*^a^ Symbol nomenclature for glycan representation was used according to: Varki A, Cummings RD, Esko JD, et al. Symbol nomenclature for glycan representation. Proteomics. 2009;9(24):5398-5399. doi:10.1002/pmic.200900708.*

**Supplementary Table 5** Differences in labeling efficiency between 2-AB and ProA for the isolated IgG sample, which were estimated based on the ratios of absolute amounts of unlabeled glycans between the samples (2-AB vs ProA) for the same initial IgG amounts (which is included in the sample names, in μg).

| sample name | FA2 | FA2[6]G1 | FA2[3]G1 | FA2G2 | FA2G2S1 | FA2G2S2 | FA2BG2S2 | AVERAGE |
| --- | --- | --- | --- | --- | --- | --- | --- | --- |
| IS-10-1 | 1.1 | 1.0 | 1.1 | 1.4 | 1.6 | 1.4 | 0.9 | 1.2 |
| IS-10-2 | 0.7 | 0.7 | 0.8 | 0.8 | 0.8 | 1.0 | 0.6 | 0.8 |
| IS-10-3 | 0.5 | 0.6 | 0.6 | 0.6 | 0.5 | 0.6 | 0.8 | 0.6 |
| IS-25-1 | 1.0 | 1.1 | 0.9 | 1.0 | 1.2 | 0.9 | 1.0 | 1.0 |
| IS-25-2 | 0.9 | 1.1 | 0.9 | 1.0 | 1.1 | 0.8 | 0.8 | 0.9 |
| IS-25-3 | 1.1 | 1.2 | 1.0 | 1.3 | 1.2 | 1.3 | 1.0 | 1.2 |
| IS-50-1 | 1.2 | 1.3 | 1.1 | 1.1 | 1.1 | 1.0 | 1.0 | 1.1 |
| IS-50-2 | 1.0 | 1.2 | 0.9 | 0.9 | 1.1 | 1.0 | 0.9 | 1.0 |
| IS-50-3 | 1.1 | 1.2 | 1.1 | 1.1 | 1.2 | 1.2 | 1.0 | 1.1 |
| AVERAGE | 1.0 | 1.1 | 0.9 | 1.0 | 1.1 | 1.0 | 0.9 | **1.0** |

**Supplementary Table 6** Differences in labeling efficiency between 2-AB and ProA for the standard IgG sample, which were estimated based on the ratios of absolute amounts of unlabeled glycans between the samples (2-AB vs ProA) for the same initial IgG amounts (which is included in the sample names, in μg).

| sample name | FA2 | FA2[6]G1 | FA2[3]G1 | FA2G2 | FA2G2S1 | FA2G2S2 | FA2BG2S2 | AVERAGE |
| --- | --- | --- | --- | --- | --- | --- | --- | --- |
| ST-10-1 | 1.0 | 1.2 | 1.1 | 0.9 | 1.0 | 1.1 | 0.6 | 1.0 |
| ST-10-2 | 2.1 | 2.4 | 2.3 | 1.9 | 2.5 | 2.0 | 1.9 | 2.2 |
| ST-10-3 | 0.8 | 0.8 | 0.8 | 0.8 | 0.9 | 0.9 | 0.8 | 0.8 |
| ST-25-1 | 1.0 | 1.2 | 1.0 | 0.8 | 1.0 | 1.0 | 0.8 | 1.0 |
| ST-25-2 | 1.3 | 1.5 | 1.1 | 1.0 | 1.2 | 1.1 | 0.9 | 1.1 |
| ST-25-3 | 0.9 | 0.9 | 0.8 | 0.8 | 1.0 | 1.3 | 0.9 | 0.9 |
| ST-50-1 | 1.0 | 1.1 | 0.9 | 0.9 | 1.1 | 1.5 | 1.0 | 1.1 |
| ST-50-2 | 1.0 | 1.2 | 0.9 | 0.8 | 1.0 | 1.0 | 1.0 | 1.0 |
| ST-50-3 | 0.8 | 0.9 | 0.8 | 0.7 | 0.8 | 0.9 | 0.7 | 0.8 |
| ST-75-1 | 1.1 | 1.4 | 1.1 | 1.0 | 1.1 | 1.1 | 1.0 | 1.1 |
| ST-75-2 | 1.0 | 1.1 | 1.0 | 0.9 | 1.1 | 1.3 | 1.1 | 1.1 |
| ST-75-3 | 0.8 | 0.8 | 0.7 | 0.7 | 0.8 | 0.9 | 0.8 | 0.8 |
| ST-100-1 | 0.9 | 1.1 | 0.8 | 0.8 | 1.0 | 1.2 | 1.1 | 1.0 |
| ST-100-2 | 1.1 | 1.2 | 1.1 | 1.1 | 1.3 | 1.5 | 1.3 | 1.2 |
| ST-100-3 | 1.0 | 1.2 | 1.0 | 1.0 | 1.2 | 1.4 | 1.1 | 1.1 |
| AVERAGE | 1.1 | 1.2 | 1.0 | 0.9 | 1.1 | 1.2 | 1.0 | **1.1** |

**Supplementary Table 7** Differences in labeling efficiency between ProA and RF-MS for the isolated IgG sample, which were estimated based on the ratios of absolute amounts of unlabeled glycans between the samples (ProA vs RF-MS) for the same initial IgG amounts (which is included in the sample names, in μg).

| sample name | FA2 | FA2[6]G1 | FA2[3]G1 | FA2G2 | FA2G2S1 | FA2G2S2 | FA2BG2S2 | AVERAGE |
| --- | --- | --- | --- | --- | --- | --- | --- | --- |
| IS-1-1 | 0.6 | 1.4 | 1.6 | 0.4 | 0.5 | 0.9 | 2.1 | 1.1 |
| IS-1-2 | 0.7 | 0.8 | 0.9 | 0.5 | 0.4 | 0.8 | 0.9 | 0.7 |
| IS-1-3 | 0.7 | 0.7 | 1.3 | 0.4 | 0.4 | 1.2 | 1.9 | 0.9 |
| IS-5-1 | 1.7 | 0.6 | 0.9 | 0.7 | 0.4 | 0.9 | 0.7 | 0.9 |
| IS-5-2 | 1.5 | 0.7 | 1.1 | 0.8 | 0.4 | 1.2 | 1.7 | 1.1 |
| IS-5-3 | 0.8 | 0.9 | 1.0 | 0.7 | 0.5 | 0.7 | 1.7 | 0.9 |
| IS-10-1 | 1.5 | 0.7 | 1.0 | 0.9 | 0.5 | 0.5 | 1.4 | 0.9 |
| IS-10-2 | 1.3 | 0.5 | 1.1 | 0.8 | 0.4 | 0.4 | 1.3 | 0.8 |
| IS-10-3 | 2.1 | 0.9 | 1.5 | 1.2 | 0.8 | 1.0 | 1.3 | 1.2 |
| IS-25-1 | 1.8 | 0.7 | 1.6 | 1.6 | 0.9 | 1.5 | 1.0 | 1.3 |
| IS-25-2 | 1.8 | 0.6 | 1.6 | 1.5 | 0.8 | 1.2 | 0.9 | 1.2 |
| IS-25-3 | 1.4 | 0.6 | 1.5 | 1.3 | 0.7 | 0.8 | 0.8 | 1.0 |
| IS-50-1 | 1.1 | 0.5 | 1.2 | 1.2 | 0.8 | 0.6 | 0.6 | 0.9 |
| IS-50-2 | 1.1 | 0.5 | 1.2 | 1.2 | 0.7 | 0.6 | 0.7 | 0.9 |
| IS-50-3 | 1.8 | 0.9 | 1.6 | 1.6 | 1.0 | 0.9 | 0.9 | 1.2 |
| AVERAGE | 1.3 | 0.7 | 1.3 | 1.0 | 0.6 | 0.9 | 1.2 | **1.0** |

**Supplmenetary Table 8** Differences in labeling efficiency between ProA and RF-MS for the standard IgG sample, which were estimated based on the ratios of absolute amounts of unlabeled glycans between the samples (ProA vs RF-MS) for the same initial IgG amounts (which is included in the sample names, in μg).

| sample name | FA2 | FA2[6]G1 | FA2[3]G1 | FA2G2 | FA2G2S1 | FA2G2S2 | FA2BG2S2 | AVERAGE |
| --- | --- | --- | --- | --- | --- | --- | --- | --- |
| IS-1-1 | 2.0 | 1.1 | 1.1 | 2.4 | 3.2 | 1.4 | 0.5 | 1.7 |
| IS-1-2 | 1.9 | 2.1 | 1.3 | 3.2 | 3.9 | 0.0 | 2.2 | 2.1 |
| IS-1-3 | 2.0 | 0.4 | 0.7 | 2.0 | 2.7 | 0.0 | 0.4 | 1.2 |
| IS-5-1 | 1.8 | 1.1 | 0.6 | 1.4 | 2.4 | 0.2 | 1.1 | 1.2 |
| IS-5-2 | 1.6 | 1.3 | 0.6 | 1.2 | 2.3 | 2.1 | 0.8 | 1.4 |
| IS-5-3 | 1.0 | 1.0 | 0.7 | 1.0 | 1.7 | 0.9 | 1.5 | 1.1 |
| IS-10-1 | 1.4 | 1.7 | 1.0 | 1.1 | 1.8 | 1.1 | 0.8 | 1.3 |
| IS-10-2 | 1.6 | 2.4 | 1.5 | 1.5 | 4.5 | 1.5 | 1.9 | 2.1 |
| IS-10-3 | 1.1 | 1.9 | 0.6 | 0.9 | 2.4 | 1.1 | 1.1 | 1.3 |
| IS-25-1 | 0.5 | 1.2 | 0.6 | 0.6 | 1.0 | 0.4 | 0.7 | 0.7 |
| IS-25-2 | 0.6 | 1.4 | 0.7 | 0.6 | 1.2 | 0.5 | 0.7 | 0.8 |
| IS-25-3 | 0.4 | 0.9 | 0.5 | 0.4 | 0.9 | 0.5 | 0.5 | 0.6 |
| IS-50-1 | 0.8 | 1.6 | 0.7 | 0.6 | 1.4 | 1.2 | 1.2 | 1.1 |
| IS-50-2 | 0.9 | 2.0 | 1.0 | 0.8 | 1.6 | 1.7 | 1.7 | 1.4 |
| IS-50-3 | 0.5 | 1.2 | 0.5 | 0.5 | 1.0 | 0.5 | 0.8 | 0.7 |
| AVERAGE | 1.2 | 1.4 | 0.8 | 1.2 | 2.1 | 0.9 | 1.0 | **1.2** |

Supplementary Table 9 Higher sensitivity of ProA and RF-MS labeled IgG N-glycans in MS enabled detection of seven additional minor N-glycan structures, which were not detectable with 2-AB.

| ProA | | RF-MS | | composition | | | | proposed structure |
| --- | --- | --- | --- | --- | --- | --- | --- | --- |
| monoisotopic mass (Da) | retention time (min) | monoisotopic mass (Da) | retention time (min) | hexsose | N-acetylhexosamine | fucose | N-acetylneuraminic acid |  |
| 1640.6914 | 10.8 | 1732.6925 | 12.7 | 4 | 3 | 1 | 0 | 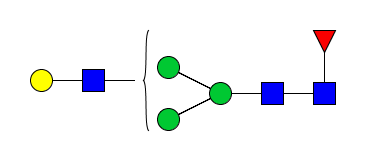 |
| 1453.6070 | 13.8 | 1545.6080 | 13.1 | 5 | 2 | 0 | 0 | 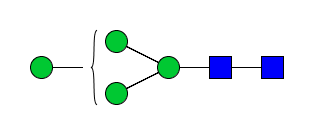  **2x** |
| 1615.6598 | 17.0 | 1707.6609 | 16.4 | 6 | 2 | 0 | 0 | 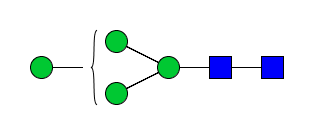  **3x** |
| 1931.7868 | 17.8 | 2023.7879 | 17.2 | 4 | 3 | 1 | 1 | 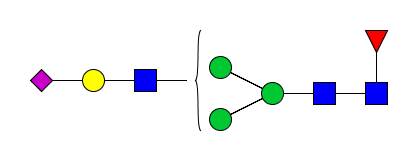 |
| 1988.8083 | 18.0 | 2080.8094 | 17.6 | 4 | 4 | 0 | 1 | 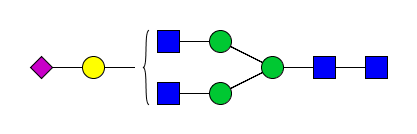 |
| 2191.8877 | 19.4 | 2283.8887 | 18.9 | 4 | 5 | 0 | 1 | 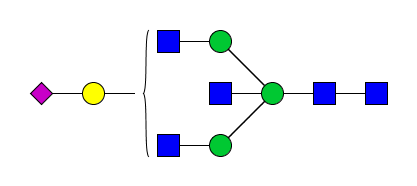 |
| 2093.8397 | 20.5 | 2185.8407 | 19.8 | 5 | 3 | 1 | 1 | 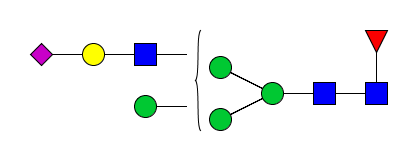 |

**
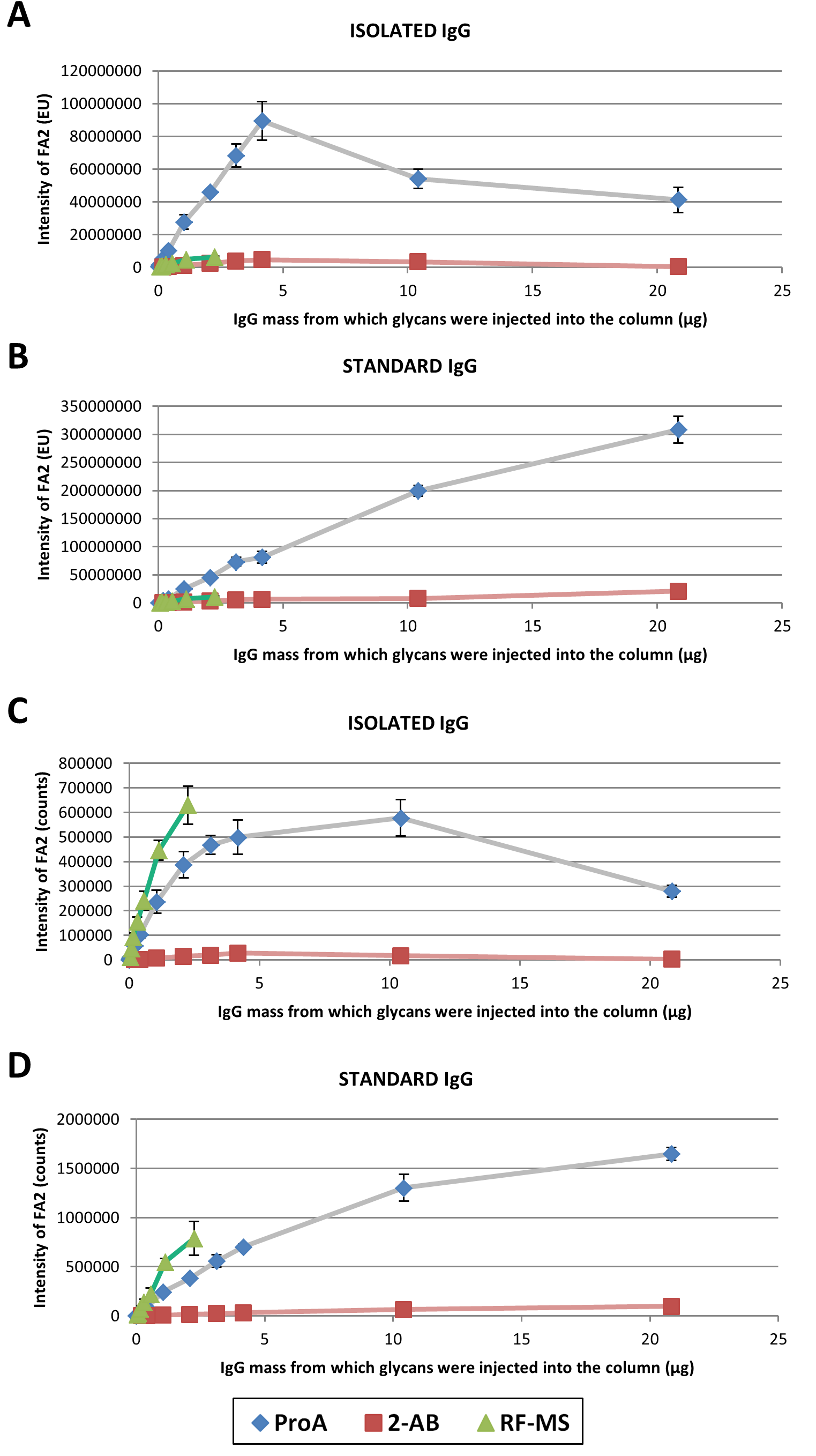
**

**Supplementary Figure 1** Signal intensity of FA2 glycan for the whole measured range, labeled with ProA (diamond), 2-AB (square) and RF-MS (triangle): for FLR signal, for isolated (A) and standard (B) IgG sample, and for MS signal, for isolated (C) and standard (D) IgG sample. Each concentration of each sample was analysed in triplicate (error bars represent standard deviation of the triplicate).


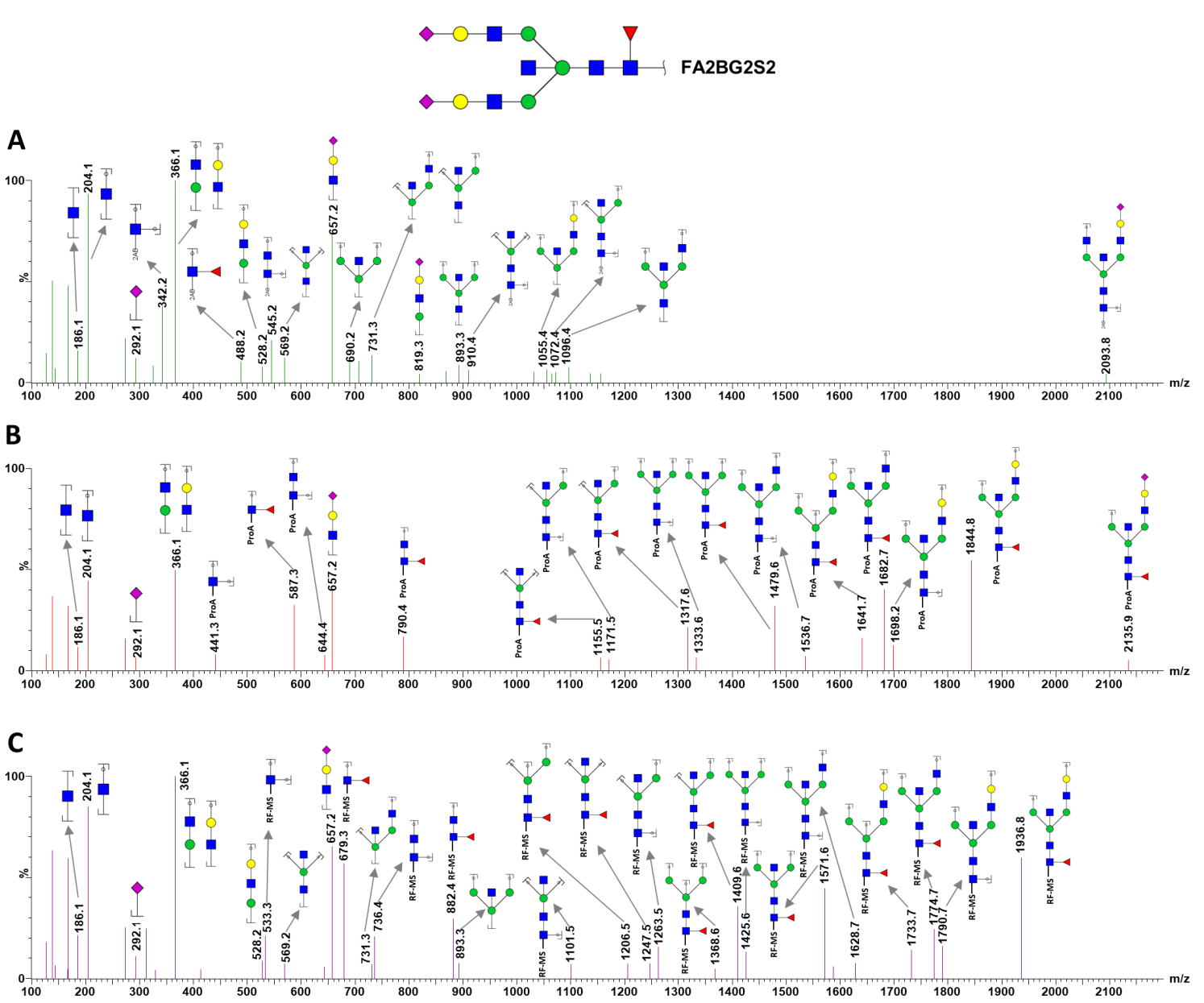


Supplementary Figure 2 Examples of ESI-QTOF-MS/MS profiles showing the fragmentation of [FA2BG2S2+2H]^2+^ labeled with: (A) 2-AB (the signal at m/z 1347.002), (B) ProA (the signal at m/z 1396.554), and (C) RF-MS (the signal at m/z 1442.555)


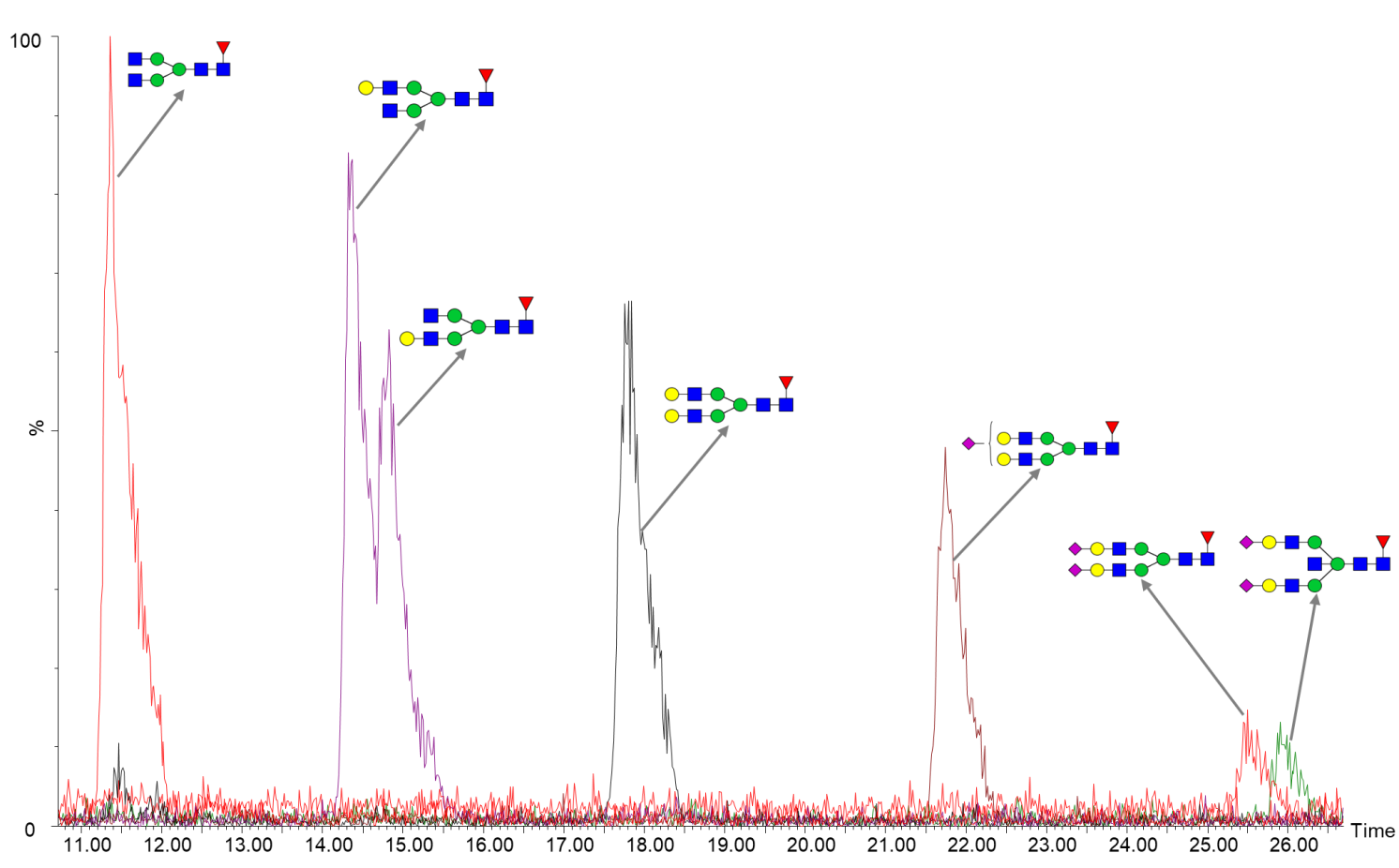


**Supplementary Figure 3** Extracted-ion chromatograms (XIC) of seven major free, unlabeled IgG N-glycans (FA2, FA2G1, FA2G2, FA2G2S1, FA2G2S2 and FA2BG2S2), which were present in each sample.
